# Inverse Method to Determine Parameters for Time-Dependent and Cyclic Plastic Material Behavior from Instrumented Indentation Tests

**DOI:** 10.3390/ma17163938

**Published:** 2024-08-08

**Authors:** Hafiz Muhammad Sajjad, Thomas Chudoba, Alexander Hartmaier

**Affiliations:** 1 Interdisciplinary Centre for Advanced Material Simulation (ICAMS), Ruhr-Universität Bochum, Universitätsstr 150, 44801 Bochum, Germany; hafiz.sajjad@rub.de; 2 ASMEC Advanced Surface Mechanics GmbH, 01109 Dresden, Germany; t.chudoba@asmec.de

**Keywords:** instrumented indentation, inverse analysis, finite element model, viscoplastic material properties, kinematic hardening, spherical indentation

## Abstract

Indentation is a versatile method to assess the hardness of different materials along with their elastic properties. Recently, powerful approaches have been developed to determine further material properties, like yield strength, ultimate tensile strength, work-hardening rate, and even cyclic plastic properties, by a combination of indentation testing and computer simulations. The basic idea of these approaches is to simulate the indentation with known process parameters and to iteratively optimize the initially unknown material properties until just a minimum error between numerical and experimental results is achieved. In this work, we have developed a protocol for instrumented indentation tests and a procedure for the inverse analysis of the experimental data to obtain material parameters for time-dependent viscoplastic material behavior and kinematic and isotropic work-hardening. We assume the elastic material properties and the initial yield strength to be known because these values can be determined independently from indentation tests. Two optimization strategies were performed and compared for identification of the material parameters. The new inverse method for spherical indentation has been successfully applied to martensitic steel.

## 1. Introduction

In recent years, the importance of the instrumented indentation approach to determine material properties has rapidly increased owing to time and cost benefits [1,2,3]. Conventional methods, such as tension, compression, or torsion tests to determine the cyclic material properties, become less attractive if the materials in the required specimen dimensions for the testing method are not available [4]. Furthermore, the conventional tests are destructive methods, which makes the indentation procedure more suitable to be applied for finished products, for example, on thin layers or for coatings [4]. In general, non-destructive methods are more resource-benign and cost-efficient. An important aspect of instrumented indentation is the ability to determine material properties conveniently at the nano, micro and macro scale [5]. For instance, Schmaling has proposed a method to determine the yield strength and work hardening rate [6] on different length scales. Furthermore, numerical simulations with spherical indentation have been used to unambiguously determine crystal plasticity parameters by using load–displacement and surface topography simultaneously [4].

Applications of depth-sensing indentation are not limited to conventional mechanical properties. For example, Faisal et al. [7] have used Vickers indentation to model the fracture toughness of thermally sprayed coatings, as the hardness of a material or finished product can be determined without affecting the quality of the material. This hardness value from indentation is used to correlate with the yield strength or tensile strength of the given material [8,9]. The relationship between material hardness and these material properties is readily available in the literature [8,9,10,11,12,13]. Furthermore, some efforts have been made to analytically correlate the hardness value with fatigue life and the S–N (Wöhler) curve [14,15,16,17]. In these approaches, either the Brinell or the Vickers hardness value is used in combination with the yield strength and the ultimate tensile strength to predict the S–N curve for different steel grades.

In conventional instrumented indentation, the specimen is loaded to a predetermined load, and then the material is unloaded. However, in the case of cyclic indentation [18,19], the indentation experiments are further extended to reload the material, and this process continues for the predetermined number of cycles. Huber [20] has numerically observed that the area of the force–displacement loop has a direct effect on the kinematic hardening fraction. In another study, Lyamkin [18] has experimentally shown that cyclic indentation has the potential to cause fatigue in the materials. As an example, they showed that the repeated application of indentation loading on austenite stainless steel at the same point caused fatigue in the material due to its cyclic elastoplastic behavior. In another study [19], cyclic indentation was combined with numerical simulations to determine the cyclic material properties, which were then used to accurately predict the uniaxial stress–strain response of the material. Furthermore, an investigation was conducted by [21] using cyclic indentation to study the impact of the indenter geometry shape in initiating film failure during nano-impact fatigue testing.

This brief overview of instrumented indentation reveals the vast application options and capability of this method to determine material properties. Furthermore, the quasi non-destructive nature of these experiments has attracted the attention of many scientists [14,15,16,17,18] who utilize instrumented indentation to predict fatigue life and other cyclic properties of materials. Some authors have suggested analytical relationships [14,15,16,17] to predict fatigue life by using the material properties determined through instrumented indentation. However, these approximations lack accuracy in predicting the fatigue properties based on the indentation hardness. Furthermore, these approaches are limited to certain metallic materials and cannot be applied to a diverse range of materials. In addition, these approaches do not take into account the microstructural features in approximating the fatigue life of a material.

Recently [19], an attempt has been made to determine cyclic material properties without considering the time-dependent viscoplastic behavior of the material. In the present investigation, this method [19] is extended to incorporate the viscoplastic behavior of the material by using a power-law creep model. Two different strategies have been presented and compared to determine the material parameters by using the inverse method. One-step and two-step optimization procedures are presented to calibrate the material response by using cyclic indentations.

## 2. Material and Experiments

### 2.1. Material Specifications

**Figure 1:**
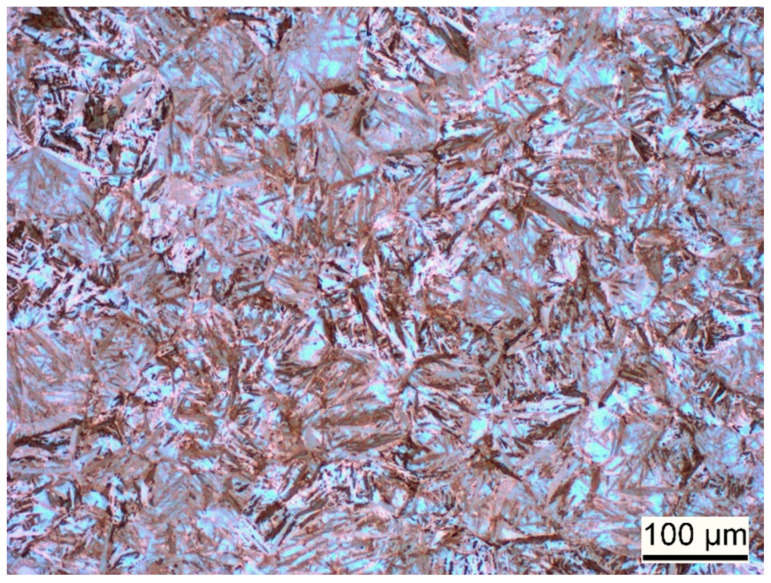
Light-microscopic image of an etched sample, showing the martensitic microstructure of the hardened and tempered steel [22].

**Figure 2:**
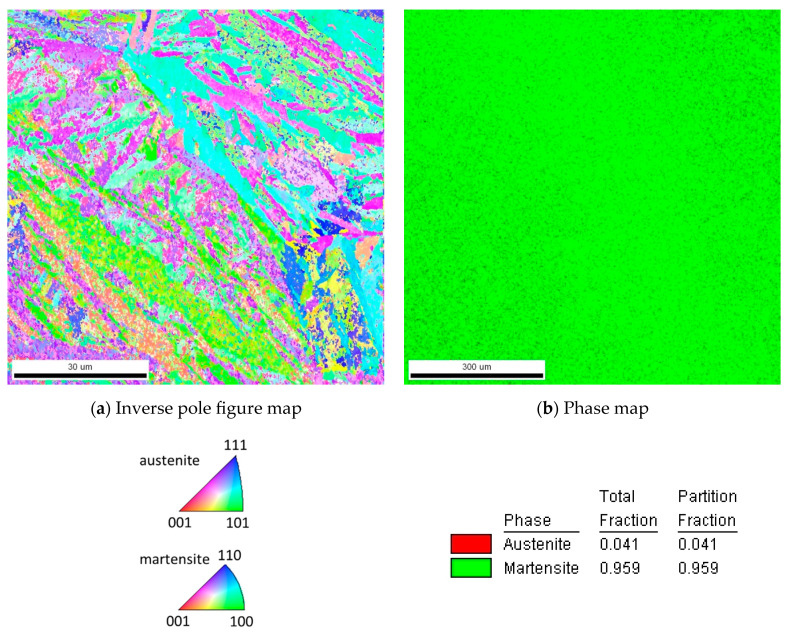
EBSD maps of the hardened and tempered steel. (**a**) Inverse pole figure map. (**b**) Phase map indicating ≈95% martensite in the microstructure [22].

The material used for the present investigation is the martensitic high-nitrogen stainless steel X30CrMoN15-1 (1.4108; AMS 5898, Energietechnik Essen GmbH, Essen, Germany). The chemical composition of this material as per the manufacturer is given in Table 1. Cyclic indentation experiments have been conducted on this material, as already described in Section 2.2. To perform validation for the predicted stress–strain hysteresis, uniaxial low-cycle strain-controlled fatigue experiments are additionally performed on this material. Figure 1 and Figure 2 show the hardened and tempered steel used for this study. Further details about the material heat treatment and experimental details of low-cycle fatigue experiments can be read in the corresponding publication [22].

### 2.2. Experimental Procedure

**Figure 3:**
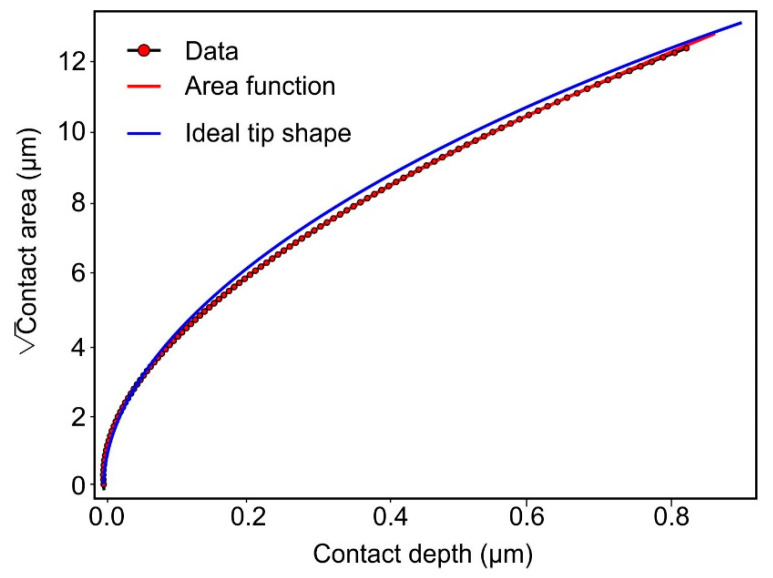
Area function as the square root of contact area over contact depth for the spherical indenter of 30.6 µm radius. The blue solid line depicts an ideal sphere with the same radius. The segment below 0.3 µm contact depth includes measurement data from fused silica and sphere, while the data above are only from fused silica.

The nanoindentation measurements were performed with a ZHN nanoindenter from ZwickRoell GmbH & Co. KG (Ulm, Germany) using a 20 N measuring head and a spherical diamond tip with a nominal radius of 30 µm. The area function was calibrated by elastic indentations with a maximum force of 2 N into fused silica and sphere single crystal with (0001) surface as reference materials. The reference values for Young’s modulus and Poisson’s ratio were E = 72 GPa, ν = 0.17 for fused silica, and E = 410 GPa, ν = 0.234 for sphere. The area function is given in Figure 3. The calculated tip radius for the depth-range below 0.3 µm, where data from both materials are available, was 30.6 µm and therefore very close to the nominal tip radius given by the manufacturer. The blue solid line depicts the area function of an ideal sphere with a radius of 30.6 µm. For a comparison with FE calculations where an ideal tip shape is assumed, it is important that the radius is not changing too much with depth. This is fulfilled for the indenter that was used for the measurements.

**Figure 4:**
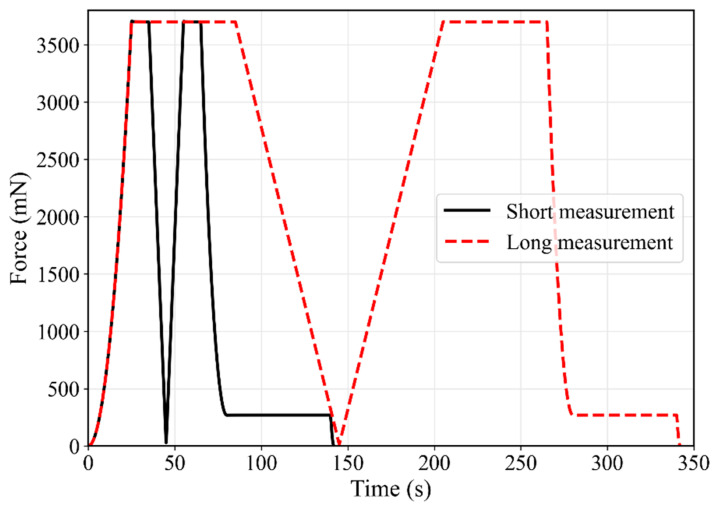
Comparison of the measurement sequences from a short and long indentation test.

The measurement sequence consisted of a loading segment with quadratic load steps, followed by a creep segment, a linear unloading close to zero force, a linear reloading to the previous maximum force, a second creep segment, a final unloading with quadratic load steps, and a hold period of 60 s at about 7% of the maximum force for the measurement of thermal drift. A maximum force of 3.7 N was chosen to reach a depth of about 15% of the indenter radius. The duration of the creep and unloading-reloading segments was varied between 10 s and 60 s. Sequences of a short and a long measurement are shown in Figure 4. The acquisition rate was 16 Hz. A thermal drift between 0.08 nm/s and 0.3 nm/s was measured during the single tests and corrected.

Five measurements were performed at all different test parameter sets, and the load–displacement curves were averaged. Only the averaged curves were further analyzed. This enabled a reduction of the influence of the grain orientation in the imprint area on the results.

Single measurements with evident differences compared to the others were excluded from averaging. The criterion for exclusion was that the maximum depth of the measurement was more than two times the depth standard deviation away from the mean depth of all measurements. A measurement uncertainty was calculated for every measurement point, based on the local standard deviation σ for force, depth, and time according to $∆x=t\sigma /\sqrt{n}$ assuming a standard distribution with a t factor for 95% confidence level (n is the number of tests).

**Figure 5:**
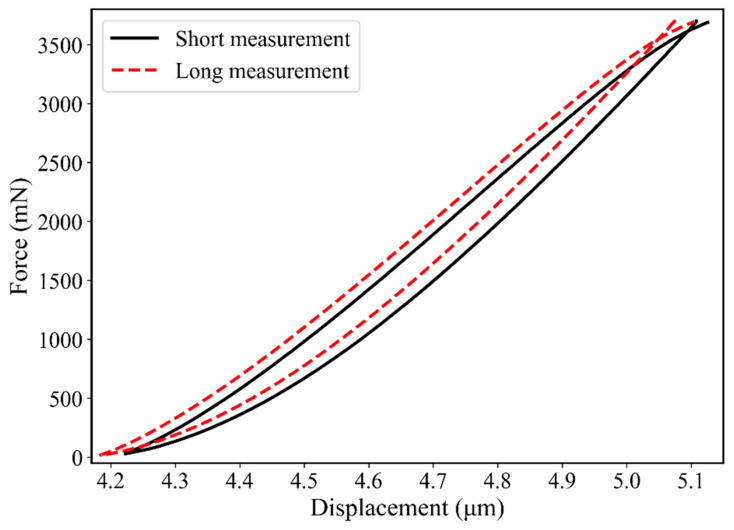
Comparison of the unloading–reloading cycles from a short (10 s for every segment) and long (60 s for every segment) indentation test.

The unloading–reloading loop of the different test sequences was analyzed in detail. The area between the curves (see Figure 5) was integrated by the instrument’s software package InspectorX and gave an energy loss. Further, the point of intersection between the unloading and reloading curve was determined. The force values of these points depend on the segment times and decrease with time.

**Figure 6:**
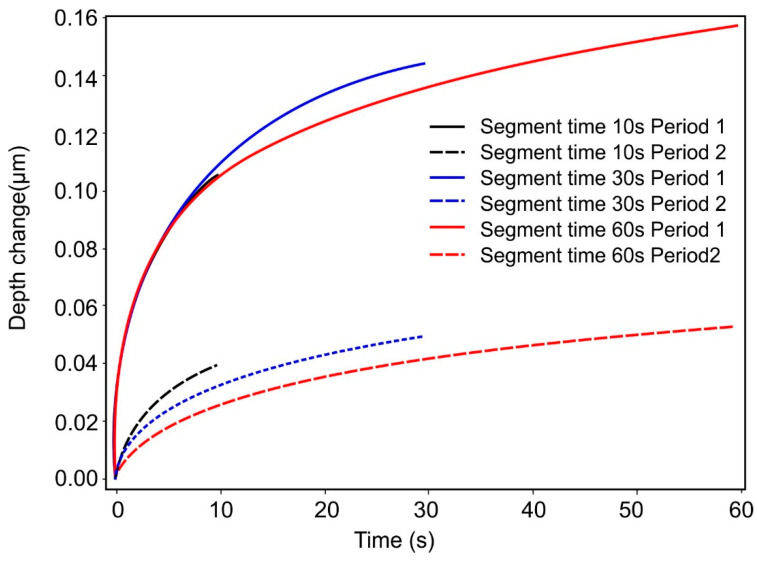
Comparison of the depth changes during the creep time for the first and second creep periods and measurements with 10 s, 30 s and 60 s segment time.

Finally, the creep segments were analyzed. In Figure 6, the changes in depth after reaching the previous maximum force are shown as a function of creep time. The duration of the first loading segment was always the same, and, therefore, the creep curves of the first holding stage should also agree within the given creep times. This was the case for segment times of 10 s and 60 s but not for 30 s. The remaining difference was the uncertainty attributed to the local differences in the sample properties. The change in depth of the second creep period was smaller because, before, there was enough time for the fading of creep effects. As expected, the depth change in the second period was smaller, as more time had passed before this second creep segment, allowing the material to relax further.

**Figure 7:**
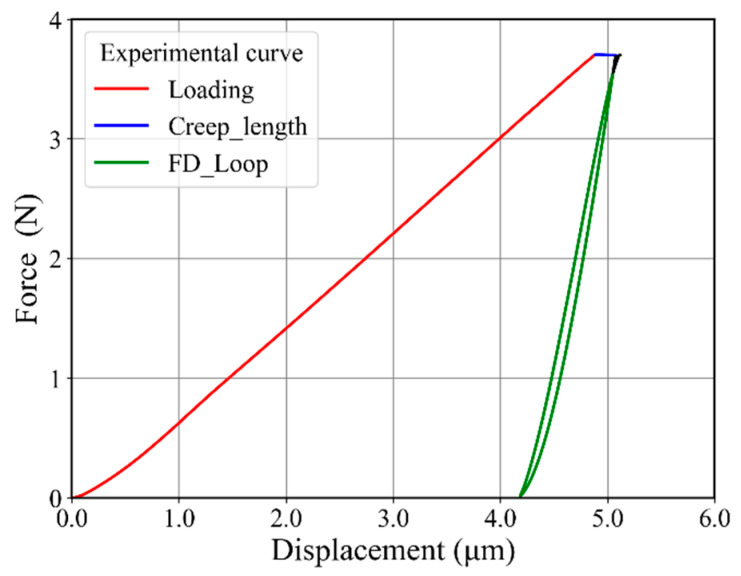
Experimental force–displacement curve for one complete cycle for indentation. The point of intersection between unloading and reloading makes a closed force–displacement loop (FD_loop).

Figure 7 shows an example of the complete force–displacement curve for one cyclic indentation, where different segments of the curve can be distinguished by the different colors. The first red part of the curve shows the loading part (3.7 N maximum force), followed by a holding stage shown with a blue solid horizontal line. The last green part of the curve shows the unloading and reloading part of the curve. An important aspect of this last part is that it makes a closed force–displacement loop just before the reloading reaches the maximum load again.

## 3. Numerical Model

**Figure 8:**
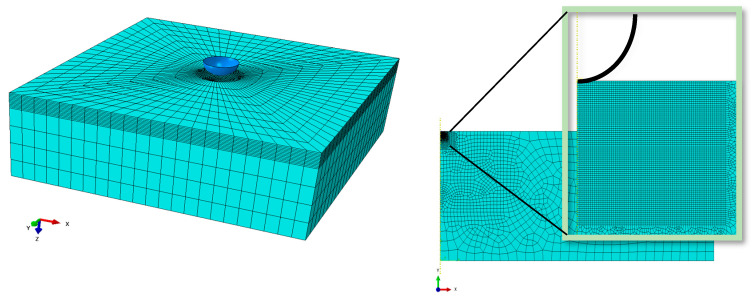
Meshing detail for a 3D model (**left**) and a 2D axisymmetric model (**right**).

**Figure 9:**
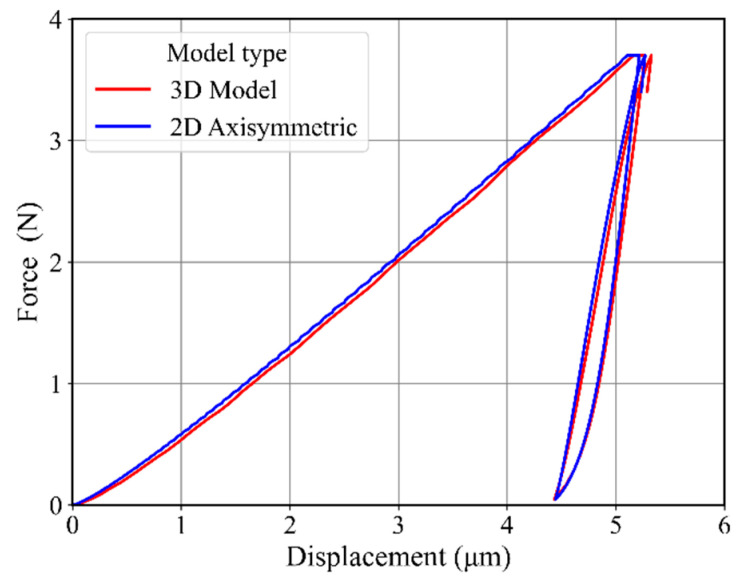
A comparison of force–displacement curves of the 3D model with 2D axisymmetric model.

The finite element models (i.e., 3D model and 2D axisymmetric model) used in the present study are shown in Figure 8. For the inverse analysis, we needed to perform many simulations during material identification optimization. The 3D model calculation takes hours to complete just one simulation. To reduce simulation time, we used a 2D axisymmetric model as shown in Figure 8 (right). For the 2D axisymmetric model, CAX4 element type with quadratic geometric order was selected. The contact between indenter and specimen was established by using a surface-to-surface contact type. The radius of the spherical-shaped Brinell indenter was kept at 30 μm, as obtained from experimental data. As the purpose of our study was to identify material parameters by using inverse analysis, the simulation time was to be as minimal as possible. This target was achieved by using the finer mesh just under the indenter tip. The mesh gradually became coarser as we moved away from the indenter, as shown in Figure 8. A sufficiently large (3.5 mm × 8 mm) 2D axisymmetric specimen was used to avoid any effects due to the smaller model size. The force–displacement curve obtained from this 2D axisymmetric model was compared with the force–displacement curve of a 3D model by using arbitrary material properties mimicking a metallic material, as shown in Figure 9.

### 3.1. Friction Effect

**Figure 10:**
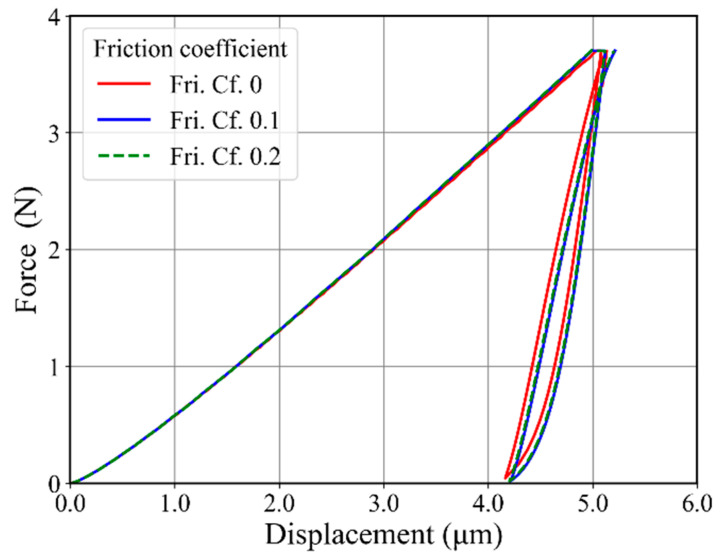
Friction coefficient study for a 2D axisymmetric model with friction coefficients 0.0, 0.1, and 0.2.

The friction effect cannot be neglected during contact problems with spherical indenters. This effect becomes prominent in the case of Brinell indentation, where the indenter makes considerable contact with the specimen during indentation. This effect was studied by choosing different friction coefficients. The comparison between friction coefficients 0, 0.1, and 0.2 is shown in Figure 10. The force–displacement curve shows a softer behavior in the case of friction coefficient 0 as compared to 0.1 and 0.2 friction coefficients. The difference between friction coefficients 0.1 and 0.2 was negligible, and after increasing the friction coefficients from 0.2 to 1.0, we did not observe any effect on the force–displacement curve for the present material. As the precise value was of minor relevance in our study, we used a friction coefficient of 0.2.

### 3.2. Rigid and Deformable Indenter

**Figure 11:**
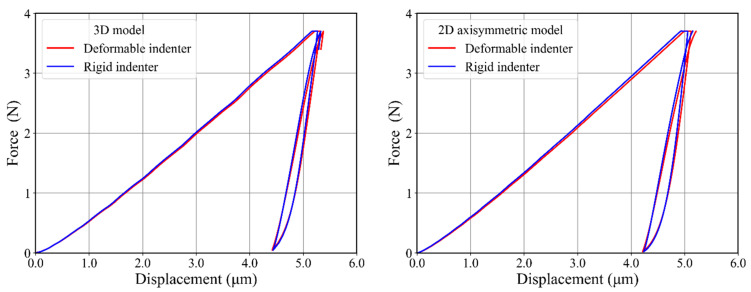
Rigid and deformable indenter comparison for 3D model (**left**) and for 2D axisymmetric model (**right**).

During the indentation experiment, some deformation of the indenter could be observed. However, this deformation was not considered by using an ideal rigid indenter during numerical simulations. To see the effect of the indenter type (i.e., rigid or deformable), we used deformable and rigid indenters during our simulations, and the results are shown in Figure 11. In the case of the 3D model, the deformable and the rigid indenter had a very slight difference in force–displacement curve, as can be seen in Figure 11. However, in the case of the 2D axisymmetric model, the force–displacement curve from the rigid indenter s slightly lagged as compared to the force–displacement curve from the deformable indenter. To approximate our simulations to experiments, we used a deformable indenter in this study.

### 3.3. Constitutive Model

Experiments show that the observed material behavior is time-dependent. This effect was particularly visible in the case of the holding stage, as can be seen the Figure 7. To avoid an overly large number of unknown parameters, we chose a simple power-law creep model for this study. In our case, the force remained constant during the holding stage. Therefore, we used a time-hardening version of power-law creep behavior in the form of

(1) $${\dot{\bar{\varepsilon }}}^{cr}={\dot{\varepsilon }}_{0}{\left(\frac{q}{\sigma_{0}}\right)}^{n}({{\dot{\varepsilon }}_{0}t)}^{m}.$$

Here, ${\dot{\bar{\varepsilon }}}^{cr}$ is the current creep rate at time t, and q is the equivalent stress according to von Mises. Furthermore, $\sigma_{0}$ is the yield stress of the material, while n, m and ${\dot{\varepsilon }}_{0}$ are the unknown material parameters which will be determined through the inverse method in the next section.

As per the definition of the von Mises yield criterion [23], the yielding in a material happens when the second deviatoric stress invariant J_2_ reaches a critical point. The mathematical formulation of this criterion f is

(2) $$f=\sqrt{\frac{3}{2}\left(S-\kappa \right):(S-\kappa )}-(\sigma_{0}+R),$$

where κ represents the back-stress tensor, S denotes the deviatoric stress tensor, $\sigma_{0}$ shows the initial yield stress before a plastic strain, and R is the drag stress. The isotropic part is captured by R, and κ controls the kinematic part. A nonlinear isotropic hardening model [24] is used to capture the strain hardening or softening. The maximum change in the yield surface due to a change in the drag stress R can be mathematically represented by

(3) $$R=Q\left(1-e^{-b\varepsilon_{eq}}\right).$$

Here Q is the stabilized stress, and the speed of its stabilization is determined by the value of *b*. The sign of Q helps to model the isotropic hardening (Q > 0) or isotropic softening (Q < 0) of the material.

Numerous kinematic hardening models are available in the literature to model the kinematic hardening behavior by using back-stress κ. For example, Armstrong and Frederick [25] suggested a nonlinear kinematic hardening model containing one back-stress term. Chaboche has improved this model by suggesting the decomposition of the single back-stress term into several back-stress terms. Thus, the Chaboche material model is quite capable of capturing the complex material response. The Chaboche kinematic hardening model with several back-stress terms can be formulated as

(4) $$\kappa =\sum_{i}^{n}\kappa_{\;i};\;d\kappa_{\;i}=\;\frac{2}{3}C_{i}d\varepsilon_{p}-\gamma_{i}\kappa_{\;i}d\varepsilon_{eq},$$

where $C_{i}$ shows the hardening moduli and $\gamma_{i}$ represents the rate of reduction of the corresponding hardening moduli as the plastic strain $d\varepsilon_{p}$ develops.

In the present study, we used the Chaboche material model, as it has already proven its ability to capture the cyclic behavior in similar studies [19,22,26,27]. Each back-stress term comprised 2 unknown material parameters, while the isotropic model had only 2 unknown material constants. The yield stress $\sigma_{0}$ value 750 MPa and the Young’s modulus 204 GPa were adopted from the experiments [22], so they were kept constant during this study.

## 4. Optimization Procedure for Material Parameter Identification

**Figure 12:**
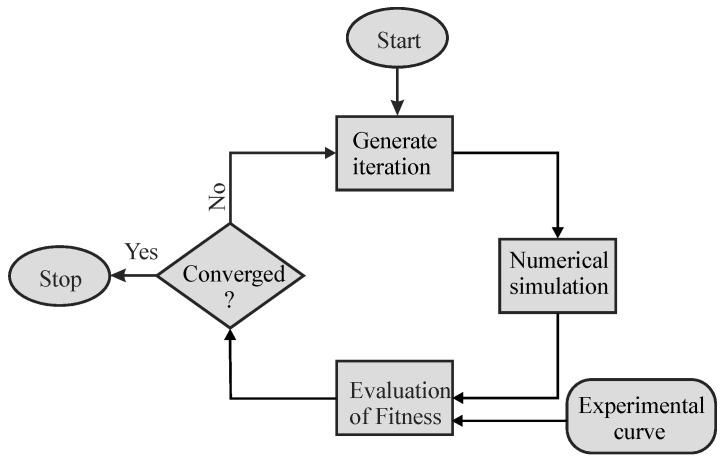
Optimization setup adopted for the iterative identification of material parameters by an inverse method based on experimental indentation data.

The material properties were determined by coupling ABAQUS simulations with the LS-Opt (DYNAmore GmbH, Stuttgart, Germany) [28] optimizer. The basic principle to determine the material parameters is to reduce the difference between the experimental curve (i.e., target curve) and the numerical curve obtained by ABAQUS simulations. These simulations were performed iteratively by varying the material parameters after each iteration. The schematic illustration of the optimization procedure is shown in Figure 12. In each iteration, the optimizer first prepares different material parameter sets to run the numerical simulation by ABAQUS. After this, the quality of fit between the target curve and the numerical curve is evaluated by using the normalized mean square error [19]:

(5) $$NMSE=\frac{1}{N}\sum_{i}\frac{{\left(E_{i}-S_{i}\right)}^{2}}{\bar{E}\bar{S}}.$$

Here $E_{i}$ and $S_{i}$ represent the experimental and simulation values, while $\bar{E}$ and $\bar{S}$ denote the average values of the respective data points. Please note that *N* is the total number of data points chosen for calculating the NMSE by selecting these points at the same force.

### 4.1. Parameter Identification by the Inverse Method

The material parameter identification was performed by using the optimization procedure explained in Section 4. In the following sections, two different approaches, named combined and 2-stage approach, respectively, have been used to determine the parameters by the inverse method. In both approaches, the range of material parameters has been fixed to the values given in Table 2.

### 4.2. Combined Parameter Identification with Two Back-Stress Terms

**Figure 13:**
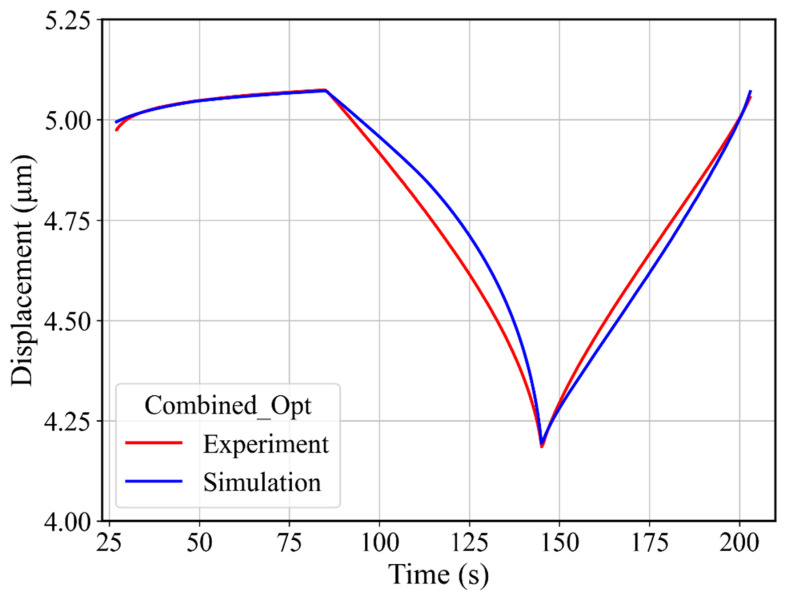
Comparison of experimental and fitted displacement–time curves from the combined optimizations.

**Figure 14:**
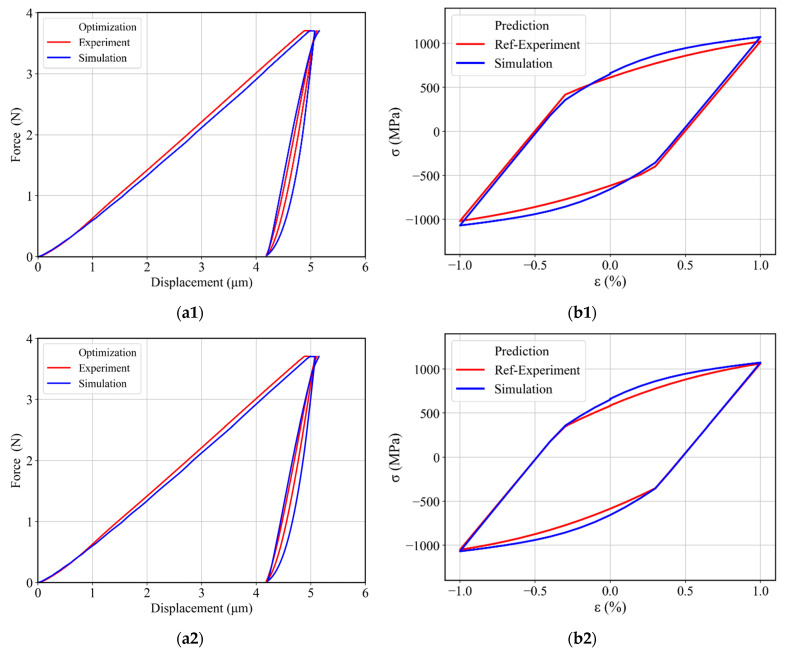

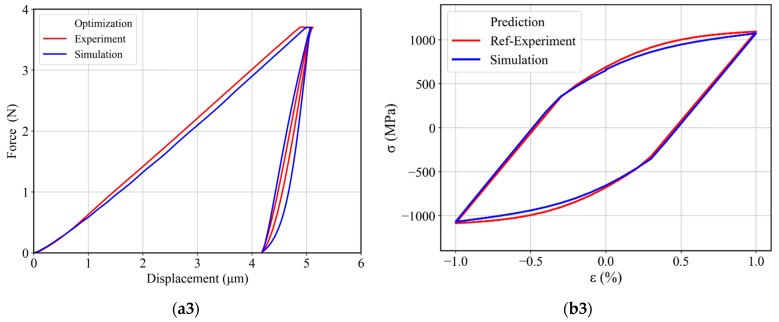
(**a**) Experimental and simulated force–displacement curves obtained after determining the material parameters by using the combined identification method with two back-stress terms: by using (**a1**) minimum (**a2**) middle and (**a3**) maximum values from Table 2 as an initial guess. (**b**) Experimental and predicted uniaxial stress–strain hysteresis by using (**b1**) Min (**b2**) Mid and (**b3**) Max identified material parameters from Table 3.

First, the displacement–time (DT) curve was extracted from the experimental data to be used as a target curve during the optimization process and to thus capture the material behavior during holding and unloading–reloading at the same time by using the respective experimental DT curve as a target curve. The target DT curve contains a holding part from 27 s (i.e., at maximum force 3.7 N) and ends at the reloading time of 203 s (Figure 13). In total, 353 points were taken into the target curve with a 0.5 s time-step in simulation and experiment. The material parameter range used in these optimizations is given in Table 2. Three optimizations were performed, each with a different starting guess, corresponding to the minimum, middle, and maximum values given in Table 2. The resulting force–displacement curves from these optimizations are plotted in Figure 14a, and the identified material parameters are given in Table 3 and Table 4. The force–displacement curves in Figure 14a provide a generally good agreement with the experimental force–displacement curves. The slightly smaller indentation depth of the loading curves in Figure 14a can be attributed partially to the difference compared to the larger experimental indenter radius of 31 µm. The deviation error, i.e., NMSE of the fitted displacement–time curves from different parameter sets is given in Table 3. Despite the generally small error values, there were some systematic deviations concerning the initial loading curve and the width of the force–displacement hysteresis loop. These systematic errors indicate that the material model itself had deficits in describing the experimental indentation process more accurately. A prediction of uniaxial stress–strain hysteresis from cyclic tensile tests was made by using each identified material parameter set, and a comparison with the experimental uniaxial stress–strain hysteresis is presented in Figure 14b for three optimizations. The prediction agrees well with the experimental stress–strain hysteresis curves, with an error margin for the dissipated work (area of the hysteresis curve) of around 4% for the best case and 8% for the worst-case optimization. Furthermore, we could observe stress deviation at ±1% strain as compared to experimental stress–strain hysteresis. For instance, in Figure 14b1, a 4.8% stress deviation is present at ±1% strain. Hence, it can be concluded that the material model parameterized based on the cyclic indentation results is capable of predicting uniaxial cyclic material behavior with an acceptable error.

### 4.3. Combined Parameter Identification with Three Back-Stress Terms

**Figure 15:**
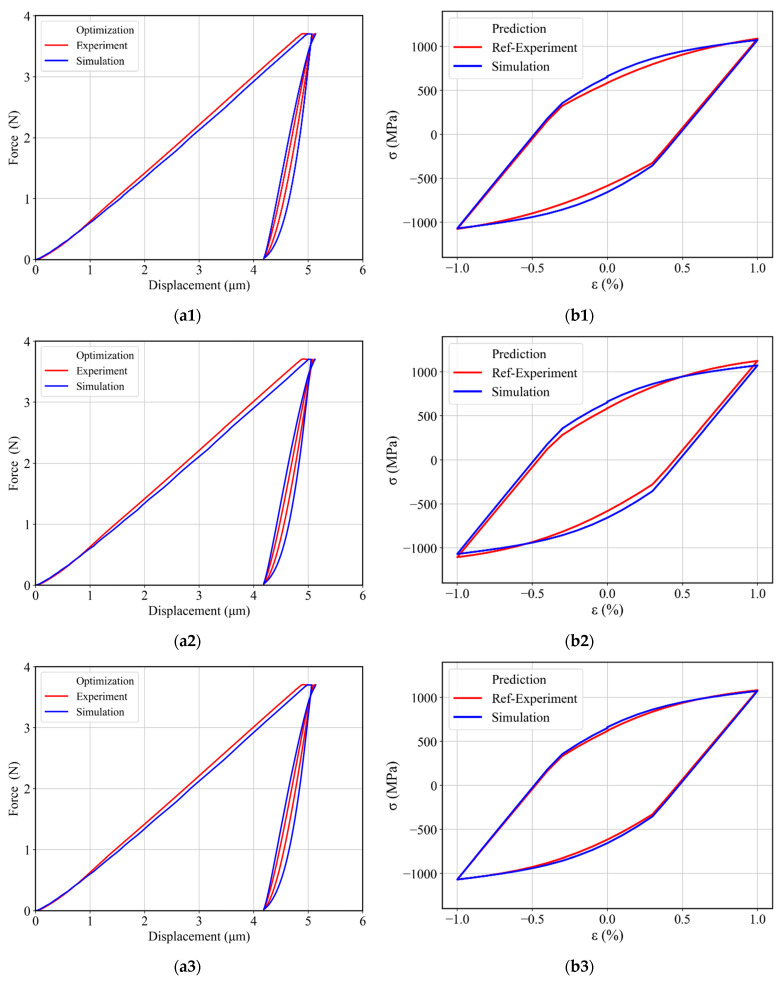
(**a**) Experimental and simulated force–displacement curves obtained after determining the material parameters by using the combined identification method with three back-stress terms: by using (**a1**) minimum (**a2**) middle and (**a3**) maximum values from Table 2 as initial guess. (**b**) Experimental and predicted uniaxial stress–strain hysteresis by using (**b1**) Min (**b2**) Mid and (**b3**) Max identified material parameters from Table 3.

To determine the optimum number of back-stress terms for our problem and to improve the uniaxial stress–strain hysteresis prediction, further optimizations were performed by using three back-stress terms instead of two back-stress terms. As before, isotropic and creep parameters were also determined during these optimizations, in addition to the three back-stress terms. Thus, 10 parameters were varied simultaneously during these optimizations by using our reference parameter range given in Table 2. Similarly to the previous optimizations with two back-stresses, the target curve for these optimizations consisted of the holding and unloading–reloading stage of the displacement–time curve. The resulting force–displacement curve for three optimizations (with the initial guess set to minimum, middle, and maximum parameters of Table 2) is plotted in Figure 15a. It can be observed that the force–displacement curves (solid blue line) from these optimizations are again in agreement with the experimental force–displacement curve (solid red line). The error of deviation (i.e., NMSE) for fitted displacement–time curves with the experimental displacement–time curve is given in Table 3. This indicates that a similar fitting can be achieved with different parameter sets, but no significant improvement for the two-back-stress optimization could be achieved. In addition to the force–displacement curves for indentations, predictions of uniaxial stress–strain hysteresis curves were also simulated by using the identified material parameters given in Table 3. It is evident from Figure 15b that all parameter sets made a reasonable prediction of uniaxial stress–strain hysteresis. The deviation in the dissipated mechanical energy of the predicted uniaxial stress–strain hysteresis curves was under 9% in the case of the min and the mid-initial guess. However, for the case of the mid parameters, a 4.6% stress deviation could be observed at ±1% strain. In the case of the max initial guess, we achieved an agreement at ±1% strain with a cyclic work error of under 5%. Thus, increasing the number of back-stress terms did not help to improve the overall uniaxial stress–strain hysteresis predictions.

Table 3 provides a comparison of the identified material parameters with two and three back-stress terms by using combined optimizations. The variance in the obtained material parameters for different optimizations was up to 50% for both, two and three back-stress terms. Only parameter C2 exhibited less than 10% variance for both methods. However, if we compare the NMSE value for both approaches, we can observe that the NMSE value remained quite consistent for three-back-stress term optimizations, and it had only 3% variance, whereas the variance in the NMSE values of the optimizations with two back-stresses was 23%. The deviation of cyclic work with two back-stress terms and three back-stress terms remained under 9% with both optimizations. Thus, considering the increased number of variables in the case of three back-stress terms and no improvement in the uniaxial stress–strain hysteresis prediction, it can be concluded that two back-stress terms are sufficient for the present inverse problem.

### 4.4. Two-Stage Material Parameter Determination

**Figure 16:**
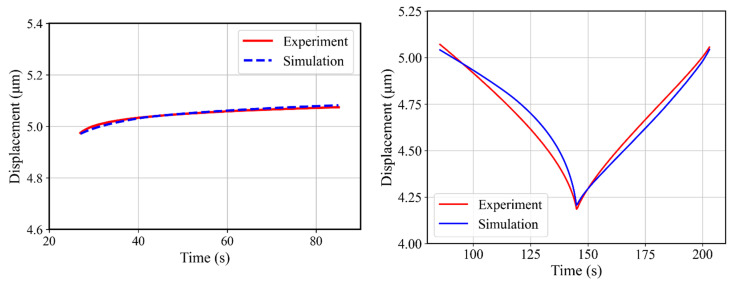
Comparison of experimental and simulated DT curves. (**left**), the holding part (**right**), and unloading–reloading part.

After determining the material parameters by adapting the combined optimization strategy, further optimizations were performed to determine the viscoplastic and hardening parameters by using a two-stage optimization strategy. In this strategy, the target displacement–time curve was divided into two parts, i.e., holding part and the unloading–reloading part. The cyclic hardening parameters were determined by using the unloading–reloading displacement–time curve as a target curve. This target unloading–reloading curve from 87 s to 203 s (Figure 16 (right)) contained a total of 233 points, starting with a time-step of 0.5 s. In this section, we only used two back-stress terms in the Chaboche kinematic hardening material model during the optimization, as a third term did not yield a significant improvement, as shown above. The target curve for obtaining Chaboche material parameters was the unloading–reloading DT curve. In agreement with the combined procedure, yield stress and Young’s modulus were kept constant during the optimization. The obtained simulated DT curve after identification of the material parameters has been compared with the respective experimental part in Figure 16; the deviation between the experimental and simulated DT was 1.0 × 10^−4^.

Having the hardening parameters, the next stage was to determine the creep parameters. The holding part was used as a target curve to determine creep parameters while keeping the rest of the parameters constant, which helped to reduce the number of unknowns in the optimization process. The holding part started at 27 s (at maximum force 3.7 N) of the experimental DT curve and ended at 85 s (Figure 16 (left)). In total, 117 points were considered in the holding target curve with a 0.5 s time-step. The material parameter range was kept the same as already used see Table 2. The simulated DT curve obtained after the optimization is shown in Figure 16. The simulated DT curve for the holding stage shows good agreement with the experimental holding stage.

**Figure 17:**
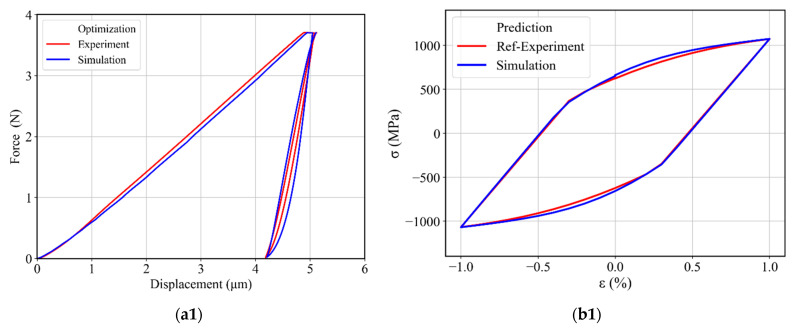

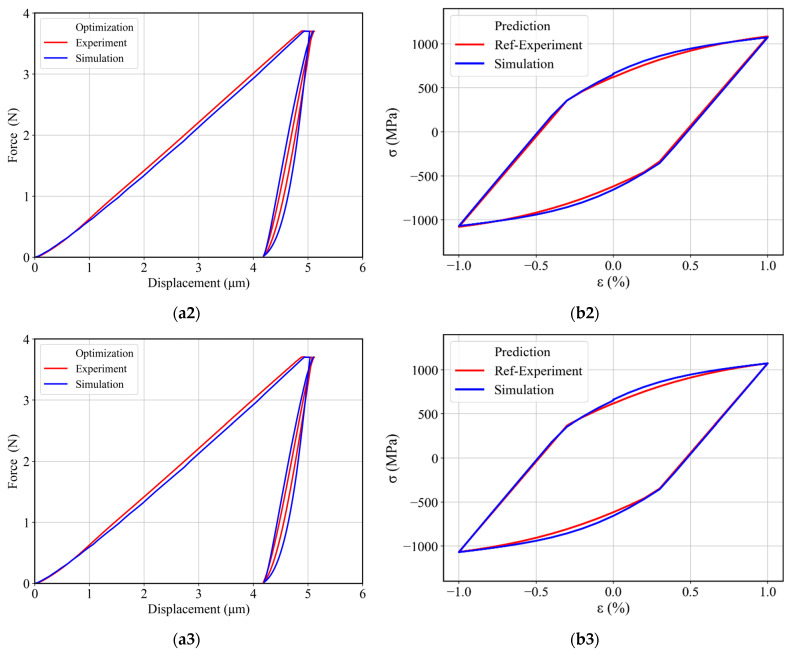
(**a**) Experimental and simulated force–displacement curves obtained after determining the material parameters by using the two-stage identification method with two back-stress terms: by using (**a1**) minimum (**a2**) middle and (**a3**) maximum values from Table 2 as an initial guess. (**b**) Experimental and predicted uniaxial stress–strain hysteresis by using (**b1**) Min (**b2**) Mid and (**b3**) Max identified material parameters from Table 4.

This two-stage parameter identification was performed with different initial guesses (i.e., starting from minimum, middle, and maximum values of parameters from Table 2), and the identified material parameters are given in Table 4. The determined creep and cyclic parameters were used to plot a complete cyclic force–displacement curve, as shown in Figure 17a. The simulated force–displacement curve depicts a rather good agreement with the corresponding experimental force–displacement curve. Furthermore, by using the same material parameters, a prediction was made for uniaxial stress–strain hysteresis with 1% total strain. The predicted stress–strain hysteresis is shown in Figure 17b together with the corresponding experimental data.

It can be observed from Figure 17a that the fitted force–displacement curves are in good agreement with the experimental force–displacement curve for all three optimizations. In addition to this, the predicted uniaxial stress–strain hysteresis from different material parameters obtained after these optimizations showed a maximum 5% cyclic work deviation from the experimental cyclic work. Furthermore, we can see an improvement at the ±1% strain for all cases, which we did not achieve in the case of combined optimizations.

Table 4 provides identified material parameters for two-stage and combined optimizations with two back-stress terms, where each approach had three material parameter sets obtained by the initial guess minimum, middle, and maximum values. From the rather significant variance in the determined material parameters, it becomes evident that there is no unique solution for the given target curve. The normalized error values (NMSE) between fitted and experimental displacement–time curves from both methods reached values of around 10^−4^. In the case of the three two-stage optimizations, the NMSE value had a 12% variance, while for the combined optimizations, this variance increased to 23%. Similarly, the kinematic hardening parameter variance for the two-stage optimizations was under 7%, while in the case of combined optimizations, the variance was more than 50%. The same trend could be seen for the rest of the parameters for both approaches. One reason for the large variance in the identified parameters for the combined approach could be a higher number of unknowns during the optimizations. The higher number of variables leads to a higher number of possible solutions. Thus, two-stage optimizations performed better than combined optimizations, as they had less variance for deviation error as well as for the identified parameter range, such that the parameters were determined with a higher certainty. In addition to this, we also observed a better prediction of uniaxial stress–strain hysteresis curves, particularly at ±1% strain.

## 5. Conclusions

A 2D axisymmetric finite element (FE) model was implemented to simulate cyclic indentation with a Brinell indenter adapting the same process parameters as used in reference experiments. To describe time-dependent and cyclic plasticity in the substrate material, a viscoplastic material model with isotropic and kinematic hardening was employed. The diamond indenter was modeled as a deformable elastic material. In the present study, we have suggested a protocol for instrumented indentation tests and a procedure for the inverse analysis of the experimental data, to obtain material parameters for time-dependent viscoplastic material behavior and for kinematic and isotropic work-hardening. Experimental indentation data for martensitic steel were determined and used for two different approaches for the optimization procedure. In this approach, two-stage and combined optimization schemes were adopted for the inverse identification of material parameters. Both optimization schemes, two-stage and combined optimizations with two back-stress terms in the kinematic hardening model, provided a good agreement of fitted force–displacement and experimental force–displacement curves. During these optimizations, the initial yield strength and Young’s modulus were assumed to be known and kept constant. The identified material parameters were used to predict cyclic stress–strain hysteresis curves obtained from uniaxial fatigue tests for the same material with only 4–8% relative error. The use of three back-stress terms did not improve the prediction quality of the uniaxial stress–strain hysteresis. Furthermore, the two-stage optimization strategy showed better prediction of uniaxial stress–strain hysteresis compared to the combined optimization. In this way, the feasibility of determining time-dependent and cyclic material properties by inverse analysis of indentation data has been demonstrated, thus significantly expanding the applicability of such methods that are currently used successfully to assess elastic material properties, yield strength, and monotonous strain hardening parameters. Hence, this work contributes to making simplified and resource-benign testing methods more powerful, allowing one to replace, or at least reduce, more time- and material-intensive conventional testing methods.

## Figures and Tables

**Table 1:** Chemical composition as provided by the manufacturer.

Element	Cr	Mo	Si	Mn	N	C	Ni	P	Al	V	Ti	Cu	S
wt.%	15.3	1.0	0.7	0.4	0.4	0.3	0.2	0.02	0.01	0.03	0.003	0.05	0.001

**Table 2:** Range of material parameters used for optimization.

Parameter	Minimum	Middle	Maximum
C_1_ (MPa)	25,000	125,000	225,000
γ_1_	100	425	750
C_2_ (MPa)	2000	3500	5000
γ_2_	0	0	0
C_3_ (MPa)	5000	77,500	150,000
γ_3_	10	255	500
Q (MPa)	−350	−187	−25
b	0.1	6	12
${\dot{\varepsilon }}_{0}$ (s^−1^)	1 × 10^−7^	5 × 10^−4^	1 × 10^−3^
n	3	7.5	12
m	−0.99	−0.5	−0.01

**Table 3:** Material parameters with two and three back-stress terms determined by varying all parameters simultaneously during optimization.

	2 Back-Stress Terms			3 Back-Stress Terms		
Parameter	Min	Mid	Max	Min	Mid	Max
C_1_ (MPa)	64,193	87,366	148,784	68,783	107,437	110,187
γ_1_	138	171	434	229	253	336
C_2_ (MPa)	3722	3616	4227	3660	3628	3567
γ_2_	0	0	0	0	0	0
C_3_ (MPa)	--	--	--	34,543	29,012	14,582
γ_3_	--	--	--	165	355	75
Q (MPa)	−252	−251	−55	−276	−275	−247
b	3.31	8.39	7.87	7.97	11.09	9.54
${\dot{\varepsilon }}_{0}$ (s^−1^)	1.54 × 10^−6^	9.46 × 10^−7^	2.87 × 10^−7^	1.86 × 10^−6^	8.39 × 10^−6^	7.67 × 10^−6^
n	10	11	9.8	8	6.5	7.7
m	−0.96	−0.92	−0.67	−0.58	−0.82	−0.91
NMSE	9.73 × 10^−5^	1.10 × 10^−4^	1.27 × 10^−4^	9.73 × 10^−5^	9.57 × 10^−5^	9.88 × 10^−5^

**Table 4:** Identified material parameters by using two-stage and combined optimization for two back-stress terms.

	Two-Stage Optimization			Combined Optimization		
Parameters	Min	Mid	Max	Min	Mid	Max
C_1_ (MPa)	87,646	94,140	90,260	64,193	87,366	148,784
γ_1_	210	221	210	138	171	434
C_2_ (MPa)	4000	4005	4011	3722	3616	4227
γ_2_	0	0	0	0	0	0
Q (MPa)	−136	−189	−107	−252	−251	−55
b	4.12	3.19	8.92	3.31	8.39	7.87
${\dot{\varepsilon }}_{0}$ (s^−1^)	7.96 × 10^−7^	1.38 × 10^−6^	1.23 × 10^−6^	1.54 × 10^−6^	9.46 × 10^−7^	2.87 × 10^−7^
n	11.83	10.7	10.95	10	11	9.8
m	−0.99	−0.99	−0.99	−0.96	−0.92	−0.67
NMSE	1.02 × 10^−4^	1.16 × 10^−4^	1.01 × 10^−4^	9.73 × 10^−5^	1.10 × 10^−4^	1.27 × 10^−4^

## Data Availability

Raw data of this research will be made available upon reasonable request.
